# Altered Expression of Vitamin D Metabolism Genes and Circulating MicroRNAs in PBMCs of Patients with Type 1 Diabetes: Their Association with Vitamin D Status and Ongoing Islet Autoimmunity

**DOI:** 10.3390/ncrna9050060

**Published:** 2023-10-07

**Authors:** Hakeemah Al-Nakhle, Ihsan Mohsen, Bashir Elnaem, Abdullah Alharbi, Ibtisam Alnakhli, Shareefa Almoarfi, Jameela Fallatah

**Affiliations:** 1 Department of Medical Laboratories Technology, College of Applied Medical Sciences, Taibah University, Al-Madinah Al-Munawaroh P.O. Box. 344, Saudi Arabia; 2 Pediatric Endocrine Division, Department of Pediatrics, Maternity & Children Hospital, King Salman Bin Abdulaziz Medical City, Madinah P.O. Box 42319, Saudi Arabia; ihsan194@yahoo.com (I.M.); bashir19medicine@gmail.com (B.E.); abdullaharbi6666@gmail.com (A.A.); ealnkhli@moh.gov.sa (I.A.); 3 Internal Medicine and Pediatrics Division, Department of Pediatrics, Maternity & Children Hospital, King Salman Bin Abdulaziz Medical City, Madinah P.O. Box 42319, Saudi Arabia; wise_net@hotmail.com; 4 Blood Bank Division, Department of Pediatrics, Maternity & Children Hospital, King Salman Bin Abdulaziz Medical City, Madinah P.O. Box 42319, Saudi Arabia; jfallat@moh.gov.sa

**Keywords:** type 1 diabetes mellitus, 1,25-Dihydroxy vitamin D, PBMCs, 1α-hydroxylase (*CYP27B1*), 24-hydroxylase (*CYP24A1*), miRNA, ongoing islet autoimmunity

## Abstract

Background: The immunomodulatory role of 1,25-Dihydroxy vitamin D3 (1,25(OH)2D3) is exerted through its interaction with the vitamin D receptor (VDR) present on pancreatic and immune cells. While a deficiency in vitamin D has been linked to Type 1 Diabetes Mellitus (T1DM), the exact molecular mechanism driving this down-regulation in T1DM is yet to be fully understood. This study aimed to decipher differences in the expression of genes associated with vitamin D metabolism in T1DM patients and to ascertain if there is a correlation between serum 1,25(OH)2D3 levels and the expression of these genes. We also sought to understand the influence of specific microRNAs (miRNAs) on the expression of vitamin D metabolism genes in peripheral blood mononuclear cells (PBMCs) of T1DM patients. Furthermore, the study delved into the potential implications of altered vitamin D metabolism genes and miRNAs on autoimmune processes. Methods: Utilizing real-time PCR, we assessed the expression profiles of genes encoding for 1-hydroxylases (*CYP27B1*) and 24-hydroxylases (*CYP24A1*), as well as related miRNAs, in PBMCs from 30 T1DM patients and 23 healthy controls. ELISA tests facilitated the measurement of 1,25(OH)2D3, GAD65, and IA-2 levels. Results: Our findings showcased downregulated *CYP27B1* mRNA levels, while *CYP24A1* expression remained stable compared to healthy subjects (*CYP27B1*, *p* = 0.0005; *CYP24A1*, *p* = 0.205, respectively). In T1DM patients, the levels of has-miR-216b-5p were found to be increased, while the levels of has-miR-21-5p were decreased in comparison to the control group. Notably, no correlation was identified between the expression of *CYP27B1* in T1DM patients and the levels of has-miR-216b-5p, has-miR-21-5p, and 1,25(OH)2D3. A significant negative correlation was identified between *CYP27B1* mRNA levels in PBMCs of T1DM and IA2, but not with GAD65. Conclusions: The study highlights there were reduced levels of both *CYP27B1* mRNA and has-miR-21-5p, along with elevated levels of has-miR-216b-5p in the PBMCs of T1DM. However, the absence of a correlation between the expression of *CYP27B1*, levels of has-miR-216b-5p, and the status of 1,25(OH)2D3 suggests the possible existence of other regulatory mechanisms. Additionally, the inverse relationship between IA2 autoantibodies and *CYP27B1* expression in T1DM patients indicates a potential connection between this gene and the autoimmune processes inherent in T1DM.

## 1. Introduction

Type 1 diabetes mellitus (T1DM) is a chronic autoimmune disease characterized by the degeneration of beta cells, resulting in insulin deficiency [1]. T1DM incidence is strongly associated with vitamin D deficiency, and 1,25(OH)2D3 can potentially prevent islet cell death and enhance insulin production. Several studies suggest that low 1,25(OH)2D3 levels may affect beta cell regulation [2]. Numerous epidemiological studies have reported a high prevalence of vitamin D deficiency among Saudi children with T1DM, indicating a strong correlation between these two factors [3]

The vitamin D metabolism pathway is controlled by various genes, including *CYP2R1*, *CYP27B1* (responsible for activation), 24-hydroxylase (*CYP24A1)* (responsible for inactivation), VDR (responsible for action), and GC. The concentration of 1,25(OH)2D3, the physiologically active form of vitamin D, is tightly regulated by both 1-hydroxylase and the catabolic enzyme 24-hydroxylase (encoded by the *CYP24A1* gene). *CYP24A1* catalyzes the hydroxylation reaction leading to the degradation of 1,25(OH)2D3, resulting in the excretion of calcitroic acid and other metabolites in the bile. *CYP24A1* expression is induced by both 1,25(OH)2D3 and 25(OH)D3, making it one of the most highly inducible genes in humans, capable of increasing its transcription by 20,000-fold [4].

Vitamin D deficiency might enhance autoimmune responses in the context of T1DM. 1,25(OH)2D3 can exert its immunomodulatory effects through genomic responses and its ability to alter gene transcription, as most human cells contain vitamin D receptors (VDR) [5]. Numerous immune cells express *VDRs* and *CYP27B1* enzymes, the synthesis of which is influenced by various immune-specific signals [6]. In autoimmune diseases, the vitamin D metabolite plays a crucial role in downregulating all mechanisms associated with adaptive immunity, inducing immunological tolerance, and promoting anti-inflammatory activity [7]. Therefore, understanding the molecular mechanism responsible for vitamin D deficiency in T1DM patients is paramount.

miRNAs are small, non-coding RNA molecules that post-transcriptionally regulate gene expression, often by preventing or triggering the degradation of mRNAs [8]. miRNAs that target genes involved in vitamin D metabolism can impact the circulating 1,25(OH)2D3 levels in individuals with T1DM. Several miRNAs have been predicted to target *CYP24A1*, but only has-miR-125b-5p has been experimentally validated [9]. Studies conducted on ovarian granulosa and breast cancer cells have shown that overexpression or antisense knockdown of has-miR-125b-5p suppresses and enhances the expression of *CYP24A1* protein, respectively [9]. In addition to its role in vitamin D catabolism, has-miR-125b-5p also targets the *VDR*. Reducing has-miR-125b-5p expression enhances the response to 1,25(OH)2D3 in melanoma cell lines [10]. Conversely, increased has-miR-125b-5p levels suppress endogenous *VDR* protein levels in MCF-7 breast cancer cells, contributing to resistance to 1,25(OH)2D [11]. While other miRNAs may potentially target VDR, only one, miR-326, has been validated in the peripheral blood lymphocytes of individuals with T1DM [12]. Considering the proposed role of 1,25(OH)2D3 in protecting against autoimmunity [13], it is reasonable to speculate that miR-326 may inhibit the immunomodulatory effects of 1,25(OH)2D3 in preventing inflammatory and autoimmune disorders. Moreover, studies have shown that miR-125a-5p is upregulated in Treg cells isolated from the lymph nodes draining the pancreas of T1DM patients. This upregulation potentially contributes to the reduced expression of its target gene, C-C Chemokine Receptor type-2 (*CCR2*) [14].

The vitamin D-activating enzyme *CYP27B1* is predicted to be targeted by multiple miRNAs, although only one has been experimentally validated. Several studies have shown that miR-21 inhibits *CYP27B1* expression in monocytes infected with Mycobacterium leprae (*M. Leprae*), thus inhibiting downstream antibacterial responses induced by vitamin D intracrine signaling [15]. Another miRNA predicted to target *CYP27B1* is has-miR-216b-5p. T1DM is associated with increased miR-216a expression in pancreatic islets, possibly as a compensatory mechanism [16]. Both miR-377 and miR-216a have been identified as early biomarkers of nephropathy in children with T1DM. Their correlation with carotid intimal thickness (*CIMT*) provides insights into the subclinical atherosclerotic processes in diabetic nephropathy [17].

Accumulating evidence suggests that miRNAs are involved in T1DM pathogenesis through multiple mechanisms, including the regulation of immune cell differentiation, development, activation, and the disruption of immune system equilibrium. Overexpression of miR-34a in diabetic mice reduces B lymphopoiesis capacity, disturbs pancreatic islet defense, and increases sensitivity to damage [18]. Differential miRNA expression also influences the production of specific T lymphocytes. Moreover, miR-26 and miR-101 have been associated with the differentiation of cells toward the T helper 1 (Th1) phenotype. Additionally, miR-21, miR-93, miR-326, and miR-31 are believed to alter T cell functions and play a role in T1DM autoimmunity [19,20].

Several miRNAs have been associated with T1DM in peripheral blood mononuclear cells (PBMCs). Patients with recently diagnosed T1DM exhibited significant downregulation of miR-21a and miR-93, which target NF-KB signaling to regulate apoptosis and inflammation [21]. Furthermore, miR-326 is overexpressed in PBMCs from T1DM patients [12,22], suggesting the involvement of miRNAs in T1DM autoimmunity as they target significant immune modulators—VDR and erythroblastosis virus E26 oncogenic homolog 1 (ETS-1). Additionally, miRNA signatures in PBMCs were correlated with autoantibodies in T1DM patients, with increased miR-326 levels correlated with antibodies against glutamic acid decarboxylase (GAD) and tyrosine phosphatase-like protein (IA2) [12,22], and reduced miR-146a levels correlated with antibodies against GAD [21]. Detecting miRNAs in PBMCs offers the advantage of using them as biomarkers for monitoring disease progression.

The cause of T1DM is not fully understood, but is believed to be the development of autoantibodies and autoreactive Th1 and cytotoxic T lymphocytes (CTLs), which cause the immune system to destroy insulin-producing pancreatic cells [1,23]. Vitamin D deficiency appears to contribute to increased activation of B cells and autoantibody production, and long-term supplementation with vitamin D led to an increase in T-regulator cells in individuals with SLE [24,25].

Here, we hypothesize that irregular miRNA expression in PBMCs from patients with T1DM might influence the downstream target genes involved in vitamin D metabolism. This could potentially play a significant role in the development of T1DM and 1,25(OH)2D3 insufficiency. Additionally, circulating miRNAs and vitamin D-related genes may affect the pathogenesis of T1DM by modulating autoimmune responses.

In this study, we aimed to ascertain whether there are differential expressions of vitamin D metabolism genes between patients with T1DM and the healthy control. Additionally, we sought to identify any potential correlations between circulating serum 1,25(OH)2D3 concentrations and the expression levels of these metabolic genes in individuals with T1DM. We further investigated the potential influence of circulating miRNAs on the expression of vitamin D metabolism genes within the PBMCs of those with T1DM. Notably, alterations in vitamin D metabolism genes and associated miRNAs could hold significance in relation to the autoimmune aspect of T1DM.

## 2. Results

### 2.1. Comparison of Demographic Characteristics and 1,25(OH)2D3 Levels of Patients with Type 1 Diabetes and Healthy Controls

Table 1 displays the demographic characteristics and 1,25(OH)2D3 levels of patients with T1DM and healthy controls. The mean age of the T1DM patients was 10.71 ± 6.072 years, compared to 9.296 ± 3.006 years for the healthy controls. There were no significant differences in age, sex, and BMI between the T1DM patients and healthy controls.

**Figure 1:**
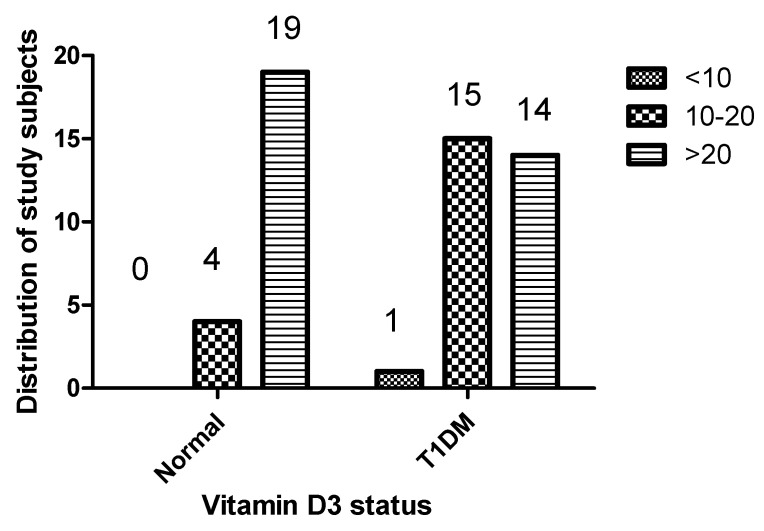
Distribution of vitamin D status among study participants with T1DM and healthy controls.

1,25(OH)2D3 serum levels were significantly lower in T1DM patients than in healthy controls, with values of 24.37 ± 17.14 ng/mL and 43.18 ± 50.89 ng/mL, respectively. A notable 53.33% of T1DM patients were classified as having insufficient 1,25(OH)2D3 levels (<20 ng/mL), whereas no deficiency was observed among the healthy controls. Specifically, the rate of 1,25(OH)2D3 deficiency was 3.33% in T1DM patients compared to zero in healthy controls, with a *p*-value of 0.0045, indicating statistical significance (Table 1). Figure 1 provides a comprehensive overview of the variance in vitamin D status between individuals with T1DM and those without, underscoring the differences in vitamin D levels between the two groups.

### 2.2. CYP27B1 mRNA Expression Is Downregulated in PBMCs from T1DM Patients Compared to Healthy Controls, While CYP24A1 Remains Unchanged

**Figure 2:**
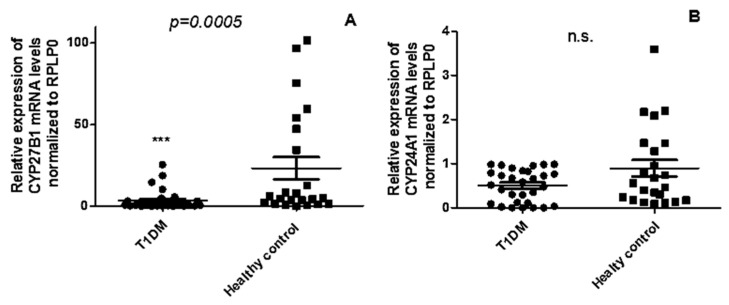
Expression levels of *CYP27B1* (**A**) are decreased in the PBMCs of patients with T1DM, whereas no significant alteration is observed in the expression of *CYP24A1*, *** *p* < 0.0005; (**B**) across N = 23, healthy control and N = 30, T1DM patients. Data are represented as mean ± SD of normalized 2−ΔCT values, relative to the expression of acidic ribosomal phosphoprotein P0 (RPLP0). The Mann–Whitney U test was employed for statistical analysis, with a significance threshold set at *p* < 0.05.

Given the decreased 1,25(OH)2D3 levels in T1DM patients compared to healthy controls, we explored the expression profile of vitamin D-metabolism genes in PBMCs of T1DM. The levels of *CYP27B1* mRNA (Figure 2A) were significantly downregulated in T1DM patients in comparison to healthy controls (*p* = 0.0005), whereas no significant changes were observed in the expression of CYP24A1(*p* = 0.205) (Figure 2B).

### 2.3. No Correlation Observed between 1,25(OH)2D3 Serum Levels and Expression Levels of CYP24A1 and CYP27B1 mRNA

**Figure 3:**
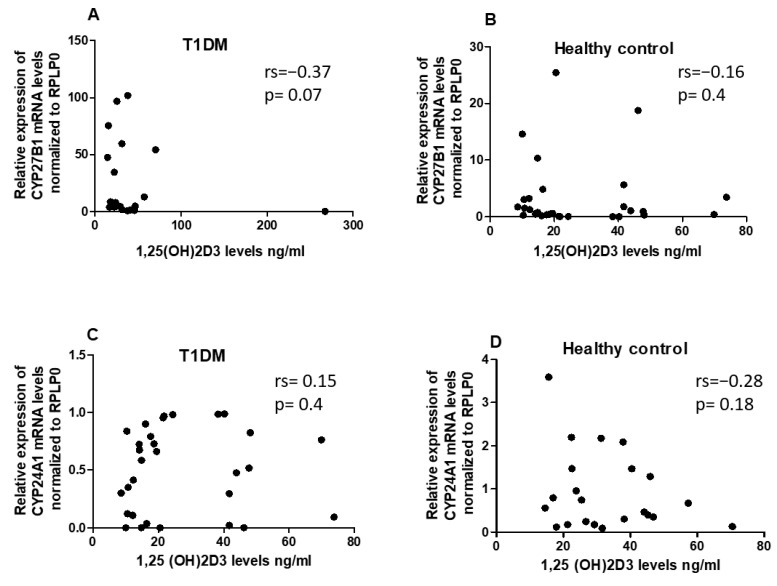
The expression levels of *CYP27B1* (**A**,**B**) and *CYP24A1* (**C**,**D**) did not exhibit any correlation with serum 1,25(OH)2D3 in both T1DM patients and the healthy control. The correlation analysis between the expression levels of *CYP27B1* and *CYP24A1* was represented by normalized 2−ΔCT values, while serum 1,25(OH)2D3 levels were reported in ng/mL. The Spearman R test was employed to determine the r-values and *p*-values, with significance set at *p* < 0.05.

To assess whether 1,25(OH)2D3 status has an impact on the expression of *CYP24A1* and *CYP27B1* in PBMCs of T1DM patients and the healthy control, we analyzed the correlation between serum 1,25(OH)2D3 levels and the expression levels of *CYP24A1* and *CYP27B1*. However, no significant correlations between 1,25(OH)2D3 and the vitamin D metabolism genes were identified in either the T1DM or the control group (Figure 3A–D).

### 2.4. Circulating Levels of hsamiR-21-5p and hsa-miR-216b-5p, but Not hsa-miR-125b-5p, Are Differentially Expressed in PBMCs of T1DM Patients Compared with Healthy Controls

**Figure 4:**
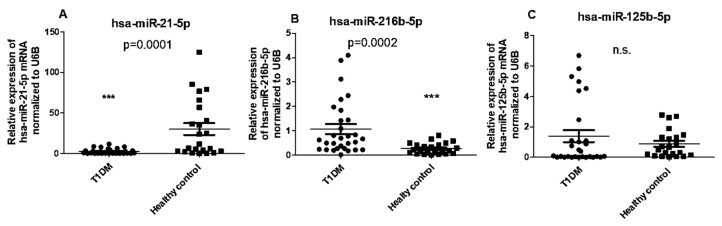
Expression of miRNA in PBMCs from patients with T1DM and healthy controls. (**A**) The level of hsa-miR-21-5p, (**B**) hsa-miR-216b-5p, and (**C**) hsa-miR-125b-5p levels in PBMCs. The error bars represent the standard deviation (SD). *** *p* ≤ 0.001 vs. controls.

Based on our findings, which indicated no correlation between serum 1,25(OH)2D3 concentrations and CYP24A1 and CYP27B1 mRNA levels in PBMCs of T1DM patients, we further investigated whether epigenetic factors such as miRNAs might play a role in regulating CYP27B1 expression. We observed a significant upregulation in the expression of hsa-miR-216b-5p, while hsa-miR-21-5p was down-regulated in PBMCs of T1DM patients compared to healthy controls. However, no significant differences in the expression of hsa-miR-125b-5p were observed between T1DM patients and controls (Figure 4A–C).

### 2.5. CYP27B1 Not Correlated with hsa-miR-216b-5p and hsa-miR-21-5p in T1DM

**Figure 5:**
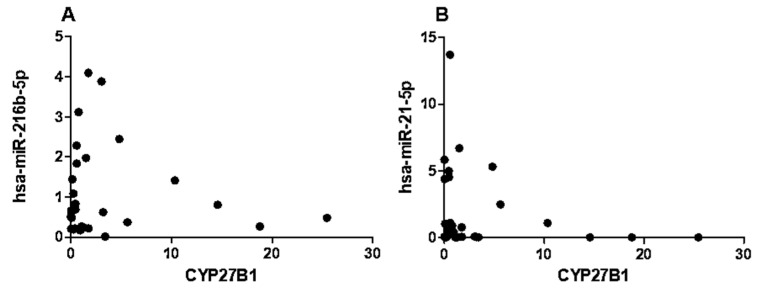
The expression levels of hsa-miR-216b-5p (**A**) and hsa-miR-21-5p (**B**) are not correlated with *CYP27B1* expression in T1DM patients. The correlation analysis is reported as normalized 2−ΔCT values. The Spearman R test was performed to evaluate R-values and *p*-values (*p* < 0.05).

Given the differential expression of hsa-miR-216b-5p, hsa-miR-21-5p, and CYP27B1 in T1DM, we performed a correlation analysis to determine whether these miRNAs were associated with *CYP27B1* expression. In T1DM patients, we observed no significant correlations between *CYP27B1* mRNA level and hsa-miR-216b-5p (*p* = 0.6, rs = 0.09, Figure 5A) or hsa-miR-21-5p (*p* = 0.2, rs = −0.23, Figure 5B).

### 2.6. Correlation between miRNAs and CYP27B1 with Islet Autoantibodies

**Figure 6:**
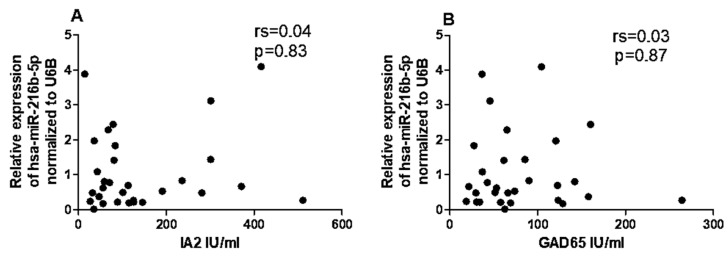
Correlation of has-miR-216b-5p expression to Islet autoantibodies within the T1DM group. (**A**) IA2, (**B**) GADA65.

**Figure 7:**
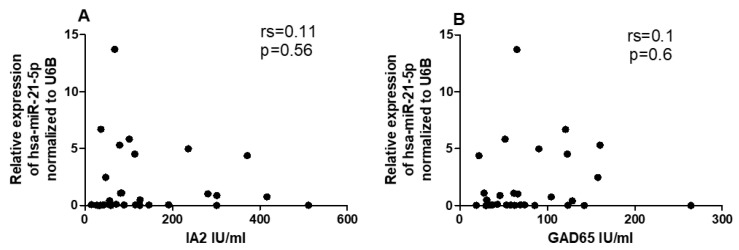
Correlation of has-miR-21-5p expression to Islet autoantibodies within the T1DM group. (**A**) IA2, (**B**) GADA65.

**Figure 8:**
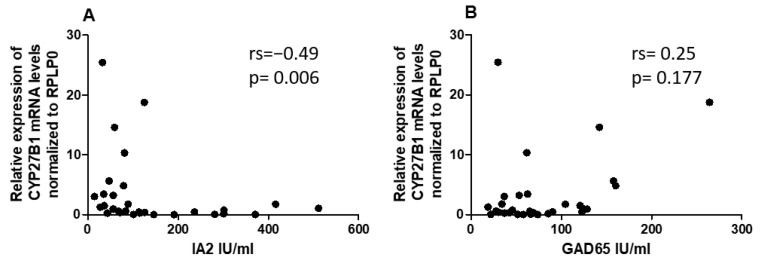
Correlation of *CYP27B1* expression to islet autoantibodies within the T1DM group. (**A**) IA2, (**B**) GADA65.

Given the differential expression of miRNAs and *CYP27B1* in PBMCs of individuals with T1DM, we investigated whether these factors could correlate with islet autoantibodies such as GADA65 and IA2. Analysis of associations between hsa-miR-216b-5p and hsa-miR-21-5p expression and titers of islet autoantibodies (GADA65 and IA2A) revealed no significant correlations (Figure 6A,B and Figure 7A,B). IA2 titers negatively correlated with *CYP27B1* (Figure 8A), whereas GADA65 titers showed no correlation with *CYP27B1* (Figure 8B).

## 3. Discussion

Vitamin D is pivotal in modulating autoimmune diseases, including diabetes. Its deficiency has been implicated in the pathogenesis of T1DM. In this context, we analyzed whether serum 1,25(OH)2D3 concentrations and vitamin D metabolic genes’ expression correlate in T1DM patients. Additionally, we investigated if miRNAs might modulate the expression of these vitamin D metabolism genes in PBMCs of T1DM, given that miRNAs targeting genes in the vitamin D metabolism pathway could influence circulating 1,25(OH)2D3 levels in T1DM. To the best of our knowledge, this is the first study linking circulating miRNA expression with alterations in vitamin D metabolism genes within PBMCs of T1DM, considering vitamin D status and ongoing islet autoimmunity.

There is a robust association between T1DM incidence and vitamin D deficiency [26]. Many epidemiological studies have underscored the prevalence of vitamin D deficiency among Saudi children diagnosed with T1DM, alluding to a potent connection between the two [3]. Our research findings revealed a heightened 1,25(OH)2D3 deficiency among T1DM patients compared to non-diabetic controls, with 53.33% of T1DM patients exhibiting reduced 1,25(OH)2D3 levels. This is congruent with prior studies highlighting the pervasive nature of 1,25(OH)2D3 deficiency among T1DM patients [27]. The relationship between vitamin D deficiency and T1DM onset—whether causative or consequential—remains elusive.

Our investigations into a potential link between vitamin D deficiency in T1DM and vitamin D metabolism genes led us to compare gene expressions in T1DM patients and healthy individuals. We noted a significant decline in *CYP27B1* mRNA levels among T1DM patients, with no observable changes for *CYP24A1*. Our results are coherent with previous findings showing attenuated *CYP27B1* mRNA levels in T1DM patients relative to controls. The implications of the *CYP27B1* gene on T1DM pathogenesis are evident as it modulates mRNA expression and influences 1,25(OH)2D3 serum levels, potentially via the −1260 C/A polymorphism [28]. However, our data did not showcase a direct correlation between *CYP27B1* mRNA levels and serum 1,25(OH)2D3. There is speculation that a correlation might exist at the protein level. Further research is needed to uncover these connections, suggesting the possibility of significant breakthroughs ahead.

Given that *CYP7B1* expression did not correlate with 1,25(OH)2D3 status in T1DM, we explored other factors potentially influencing its expression. Prior research suggests epigenetic modifications, such as miRNAs, might impact *CYP27B1* gene expression. Given the significant role of miRNAs in T1DM pathogenesis and *CYP27B1* expression regulation in other tissues [29], we probed the potential of miRNA in modifying *CYP27B1* expression. Bioinformatic predictions posited that has-miRNA-216b-5p and has-miRNA-21-5p could target the 3′ UTR of *CYP27B1*, while has-miRNA-125b-5p might target *CYP24A1′*s 3′ UTR. The expressions of influencing miRNAs, governed by factors like BMI and age, were consistent across both groups in our study. Notably, our findings indicated an upregulation of has-miR-216b-5p and a downregulation of has-miR-21-5p, with no alterations for has-miR-125b-5p. Prior research corroborates the differential expression of has-miR-21-5p and has-miR-216b-5p in T1DM [16,21,30,31]. Interestingly, while our results showcased differential expression of specific miRNAs, consistent with previous studies, they did not directly correlate with *CYP27B1* in PBMCs of T1DM. This raises the tantalizing possibility of other underlying mechanisms, perhaps genetic polymorphism or insulin resistance, playing a role in modulating *CYP27B1* expression.

While the crucial role of miRNAs in preserving immune equilibrium is apparent, the perturbed expression of specific miRNAs and its subsequent contribution to autoimmune diseases introduces new avenues of exploration [32]. The accumulating evidence pointing towards miRNAs’ involvement in T1DM pathogenesis—particularly in domains like immune responses and beta-cell metabolism—poses thought-provoking questions [33]. Might other unidentified miRNAs be just as critical, if not more so, in influencing T1DM onset? The prevailing research, emphasizing miRNAs’ role in immune stability and the negative impact of their anomalous expression on autoimmunity, raises queries about potential therapeutic interventions harnessing miRNA regulation [32,33,34]. With multiple studies documenting shifts in miRNA expression linked to T1DM pathology, one cannot help but speculate if reversing these alterations could mitigate disease progression [21] [12,22]. Remarkably, the diminished expression of miR-21 and miR-93 in T1DM patient PBMCs, vital to inflammatory and apoptotic pathways, hints at potential therapeutic targets [21]. Given the consistent reduction in has-miR-21-5p in our study and others, can enhancing its expression offer therapeutic benefits? Further comprehensive studies might unravel the potential opportunities within miRNA regulation in T1DM.

Several pivotal antibodies can be detected in T1DM patients’ years before the onset of the disease, notably antibodies against IA-2, IA-2b, and GAD. Intriguingly, research suggests that miRNAs may play a crucial role in the biosynthesis of specific auto-antibodies, as a cluster of 32 miRNAs has been found to modulate the expression pattern of these T1DM-related auto-antibodies [35]. However, our findings reveal no correlation between miRNAs and auto-antibodies.

Subsequently, we sought to understand the potential role of reduced *CYP27B1* expression on the presence of autoantibodies in patients with T1DM. This investigation entailed correlating *CYP27B1* mRNA levels with specific autoantibodies, notably IA2 and GAD65. We discovered a negative association between *CYP27B1* mRNA levels and IA-2 autoantibodies, suggesting a compelling possibility: the *CYP27B1* gene might play a pivotal role in the autoimmune cascade characteristic of T1DM. Moreover, previous studies have revealed associations between polymorphisms in codon 416 of the vitamin D-binding protein gene and autoimmune markers of T1DM, particularly IA2 antibodies [36]. Given these findings, it raises the question: Could the intricate interplay of genetic factors, miRNAs, and vitamin D metabolism be fundamental to the complex etiology of T1DM? Future research endeavors are poised to unravel this intricate network of interactions and illuminate the underlying mechanisms of the disease.

The clinical implications of this study underscore the profound significance of 1,25(OH)2D3 levels and related metabolic gene expressions such as *CYP27B1* in the early detection and monitoring of the disease. Furthermore, altering miRNA expression in T1DM may provide promising targets for future pharmaceutical interventions, enhancing the overall management and treatment avenues. Additionally, as the study reveals correlations between *CYP27B1* expressions and T1DM, it emphasizes the potential of personalized medicine, wherein treatments could be tailored to the unique genetic profiles of patients. This could revolutionize how T1DM is managed, shifting from generic treatments to more individual-centric ones. Clinicians might be encouraged to adopt a more holistic approach to managing T1DM, considering an array of factors beyond just the traditional metrics of glucose and insulin.

The study presented here offers several commendable strengths, making it a valuable contribution to the current scientific understanding. Firstly, its pioneering nature cannot be understated; this research appears to be the initial foray into connecting circulating miRNA expression with alterations in the vitamin D metabolism genes, specifically in PBMCs of T1DM, thereby potentially bridging an existing knowledge gap. This comprehensive study approach stands out, as it delves into the surface-level investigation of serum 1,25(OH)2D3 levels and ventures deeper to uncover the underlying genetic and epigenetic mechanisms that might influence these levels in T1DM patients. Furthermore, the depth of analysis is worth noting. The research did not merely stop at identifying genes with altered expressions but extended its exploration to establish correlations with other physiological markers, such as autoantibodies. Considering the burgeoning interest in the role of vitamin D in T1DM and the increasing significance attached to miRNAs in various disease processes, this study is timely and relevant.

However, like all scientific endeavors, this study is not devoid of limitations. A significant constraint is that the gene expression was gauged only at the mRNA level. While this offers a snapshot, it might not always mirror the protein levels or biological activity, which are critical for a complete understanding. The sample size, being limited, raises concerns about the robustness and generalizability of the findings. Moreover, while the study adeptly uncovers various correlations, the age-old causality conundrum remains. It is yet to be definitively ascertained whether vitamin D deficiency is a consequence or a precursor to T1DM. Not to mention, there might be lurking variables.

In light of the above, it becomes evident that while this study paves the way, there is a long journey ahead. More extensive cohort studies, augmented with in vitro or animal model experiments, must validate and expand on these preliminary findings. Evaluations at the protein level, coupled with functional assays, might be the key to unlocking a more profound mechanistic understanding of the processes at play.

## 4. Materials and Methods

### 4.1. Ethics and Consent

This research project underwent a rigorous review and received approval from the Institutional Review Board at the General Directorate of Health Affairs (GDHA) in Madinah (IRB Number: 276). The GDHA, an official government ethics committee, issues ethical approvals for human research on behalf of the Ministry of Health (MOH). All studies involving human participants were conducted in compliance with the ethical guidelines set forth in the Declaration of Helsinki. Every participant provided written informed consent to participate in the study.

### 4.2. Study Design, Recruitment of T1DM Patients, Healthy Control, Inclusion and Exclusion Criteria

In this case–control study, patients with T1DM were evaluated for symptoms of diabetes and a casual plasma glucose concentration of 7.0 mmol/L or a 2-hour post-load glucose concentration of 11.1 mmol/L during an oral glucose tolerance test, based on the criteria set by the American Diabetes Society. Age-matched control children were selected from the Maternity and Children’s Hospital.

Inclusion criteria for cases included an age group of 0 to 16 years and diagnosed cases of T1DM with classical symptoms (polyuria, polydipsia, and polyphagia) along with a random plasma glucose of ≥200 mg/dL. Patients with pancreatic disease, hepatic disease, renal disease, bone diseases, malignancy, and any history of drug use such as calcium and vitamin D were excluded from the cases.

For healthy controls, individuals with blood sugar within normal limits, normal growth and puberty range, and no endocrine abnormalities or autoimmune conditions were included. Subjects with pancreatic disease, hepatic disease, renal disease, bone disease, malignancy, and any history of drug use such as calcium and vitamin D were excluded from the control group.

Data, including demographic data, medical history, comorbidities, and medications, were collected for each patient from the medical records at the Maternity and Children’s Hospital.

### 4.3. Isolation of Peripheral Blood Mononuclear Cells (PBMCs)

Blood samples (5 mL) were collected from both T1DM patients and healthy controls into tubes containing EDTA and were diluted 1:1 in PBS. Following this, density gradient separation of the diluted samples was carried out using Hypaque-Ficoll medium (Innotrain, Kronberg, Germany). PBMC samples were isolated through centrifugation of the Hypaque-Ficoll gradient, and fresh PBMCs were immediately utilized for RNA extraction.

### 4.4. Total RNA and miRNA Extraction from PBMCs

Total RNA, including miRNA, was extracted from the purified PBMCs using the Qiagen miRNeasy Mini Kit (Qiagen, Valencia, CA, USA), following the manufacturer’s protocol. The purity and concentration of RNA were determined by obtaining OD260/280 readings using a dual-beam UV spectrophotometer (Eppendorf AG, Hamburg, Germany).

### 4.5. Reverse Transcription and Quantitative Real-Time PCR

Complementary DNA synthesis from miRNA samples was carried out using the miScript II RT Kit and HiSpec Buffer (Qiagen), following the manufacturer’s instructions. Quantitative real-time PCR (qRT-PCR) was conducted using the miScript SYBR Green PCR Kit (Qiagen). The 25 μL reaction included 1 μL of cDNA, forward and reverse primers at optimized concentrations, and RNase-free water to make up the volume. The PCR was set up in a 96-well plate with triplicates, under a UV-irradiated hood on an ABI 7700HT PCR machine (Applied Biosystems 7500 Fast Real-Time PCR System) (Applied Biosystems, USA). Minus RT and no-template controls were included for each assay. The cycling conditions were: 50 °C for 2 min, 95 °C for 10 min followed by 40 cycles of 95 °C for 15 sec and 60 °C for 60 s, and a melt curve stage (95 °C for 15 s, 60 °C for 1 min, 95 °C for 15 s). All primer sequences for the selected miRNAs and vitamin D-related genes for real-time RT-PCR analyses are listed in Table S2.

Results from the real-time PCR were computed using the equation [2ˆ(−∆Ct)]. The expression levels of *CYP27B1* and *CYP24A1* mRNA were normalized to the housekeeping gene, acidic ribosomal phosphoprotein P0 (*RPLP0*). For miRNAs, hsa-miR-U6B was used as a housekeeping gene to normalize all Ct values. Ct values were determined using the SDS software v.2.1, with manual baseline settings applied to fix the same threshold for both target miRNAs and reference genes.

### 4.6. Serum Sample Collection and 1,25(OH)2D3 Level Measurement

A 3 mL blood sample was centrifuged at 1500× *g* for 10 min to obtain serum samples from both patients and controls. These serum samples were then frozen at −80 °C until required for analysis. Serum 1,25(OH)2D3 levels were measured using a vitamin D Kit (IDS, Boldon, UK) in a consistent laboratory setting. A concentration of 20 ng/mL or higher was considered a normal level of 1,25(OH)2D3. Concentrations between 10 and 20 ng/mL were classified as 1,25(OH)2D3 insufficiency, while levels below 10 ng/mL were categorized as 1,25(OH)2D3 deficiency.

### 4.7. Serological Analysis

Anti-GAD65 and anti-IA2 antibodies were assessed using enzyme immunoassay (ELISA) with Medizym commercial kits (Berlin, Germany). The detection of antibodies was performed semi-quantitatively, referencing a value of 5 IU/mL for GAD65 and 10 IU/mL for IA2. HbA1c levels were determined using a commercially available automatic system (DCA 2000, Bayer Diagnostics, Tarrytown, NY, USA).

### 4.8. Target Gene Identification and Bioinformatic Analysis

A comprehensive search of the literature was undertaken to identify a particular set of miRNAs whose expression has been previously linked with T1DM and are thought to influence the vitamin D signaling pathway. Our study concentrated on three miRNAs, namely hsa-miR-125b-5p [30,31,37], has-miR-216b-5p [16,17],and has-miR-21-5p [38,39], which have been significantly dysregulated in serum, plasma, and PBMCs of T1DM patients, as identified using bioinformatics tools (TargetScan Human, available at http://www.targetscan.org) accessed on 22 December 2022. Based on the extant literature encompassing in vitro studies, we selected miRNAs potentially interacting with the 3′UTR sequences of *CYP24A1* and *CYP27B1* (refer to Supplementary Table S1). Among several miRNAs that might interact with the *CYP24A1* 3′UTR sequence, we chose hsa-miR-125b-5p. For the *CYP27B1* 3′UTR, hsa-miR-216b-5p and hsa-miR-21 were selected. The involvement of these miRNAs in regulating *CYP27B1* expression was substantiated by in vitro studies.

### 4.9. Statistical Analysis

Statistical analyses were conducted using GraphPad Prism 6.0 software (GraphPad Software, Inc. San Diego, CA USA). Clinical parameters are represented as mean ± standard deviation (SD). To compare statistical significance between the two groups (T1DM vs. the healthy control), the unpaired t-test or Mann–Whitney U test was employed. Expression of miRNA or vitamin D-related genes is denoted as mean ± standard error. To identify correlations between miRNA expression and mRNA of vitamin D-related genes in all participants, Pearson or Spearman correlation testing was utilized. A two-tailed *p*-value (*p*) < 0.05 was deemed statistically significant for all statistical tests.

## 5. Conclusions

In conclusion, this study explored the role of vitamin D and its metabolic genes in T1DM, focusing on the correlation between serum 1,25(OH)2D3 concentrations and vitamin D related gene. A substantial association between vitamin D deficiency and T1DM incidence was noted in the investigated patients. The study found a significant decrease in *CYP27B1* mRNA levels in T1DM patients, which is crucial for vitamin D metabolism, but no direct correlation with serum 1,25(OH)2D3 was observed. Further, the research delved into the impact of miRNAs on vitamin D metabolism genes, revealing differential expression of certain miRNAs, yet without a direct correlation to *CYP27B1* in T1DM patients. This highlights other potential mechanisms influencing gene expression.

The study also found no direct correlation between miRNAs and T1DM-related auto-antibodies but discovered a negative association between *CYP27B1* mRNA levels and IA-2 autoantibodies, indicating a potential role of the *CYP27B1* gene in T1DM autoimmunity. The research prompts inquiries about the intricate interactions between genetic factors, miRNAs, and vitamin D metabolism in T1DM’s complex etiology. Future research is needed to unravel this complexity and the potential therapeutic interventions based on miRNA regulation and vitamin D metabolism in T1DM.

## Figures and Tables

**Table 1:** Demographic and clinical features of T1DM patients and the healthy control groups.

Variable	Control Group, *n* = 23	T1DM Group, *n* = 30	*p*-Value
HbA1c (%)	5.126 ± 0.4158	8.957 ± 1.353	<0.0001
IA2 (IU/mL)	0	140.4 (14.45–511.0)	-
GAD65 (IU/mL)	2.62 (0.4–4.7)	79.55 (18.77–264.3)	-
Age (years)	9.296 ± 3.006	10.71 ± 6.072	0.3098
Sex: male/female	11/12	15/15	-
BMI	21.86 ± 3.250	22.31 ± 4.248	0.6775
1,25(OH)2D3 (ng/mL)	43.18 ± 50.89	24.37 ± 17.14	0.0045

Data are presented as either medians with interquartile ranges or as means ± standard deviations. Abbreviations: BMI, body mass index; T1DM, type 1 diabetes mellitus; HbA1c, glycated hemoglobin; GAD, glutamic acid decarboxylase; IA2, islet antigen-2, a tyrosine phosphatase-like protein. Reference values are 5 IU/mL for GAD65, and 10 IU/mL for IA2; 1,25-Dihydroxy vitamin D3 (1,25(OH)2D3); *p* < 0.05 for comparison between T1DM and control groups.

## Data Availability

The data is unavailable due to privacy and ethical restrictions.

## Supplementary Materials

The following supporting information can be downloaded at: https://www.mdpi.com/article/10.3390/ncrna9050060/s1, Table S1. miRNAs prediction for *CYP24A1* and *CYP27B1* using Target scan and miRTarBase; Table S2. Primers sequences for the Vitamin D metabolism genes of the related miRNAs for real-time RT-PCR analyses.
