# Supplementary material for: Altered Expression of Vitamin D Metabolism Genes and Circulating MicroRNAs in PBMCs of Patients with Type 1 Diabetes: Their Association with Vitamin D Status and Ongoing Islet Autoimmunity
Source: Noncoding RNA. 2023 Oct 7;9(5):60. doi: 10.3390/ncrna9050060 (PMC10609170; doi:10.3390/ncrna9050060)
Supplement: Supplementary file 1 [file ncrna-09-00060-s001.zip › ncrna-2615105-supplementary.pdf]

Table S1. miRNAs prediction for *CYP24A1* and *CYP27B1* using Target scan and miRTarBase

1-CYP24A1

A-miRTarBase

ID	Species (miRNA)	Species (Target)	miRNA	Target	Validation methods: Strong evidence: Reporter assay	Strong evidence: Western blot	Strong evidence: qPCR	Less strong evidence: Microarray	Less strong evidence: NGS	Less strong evidence: pSILAC	Less strong evidence: Other	Less strong evidence: CLIP-Seq	Sum	# of papers
MIRT004408	Homo sapiens	Homo sapiens	hsa-miR-125b-5p	CYP24A1	✔	✔	✔				✔		4	2

B-Target scan

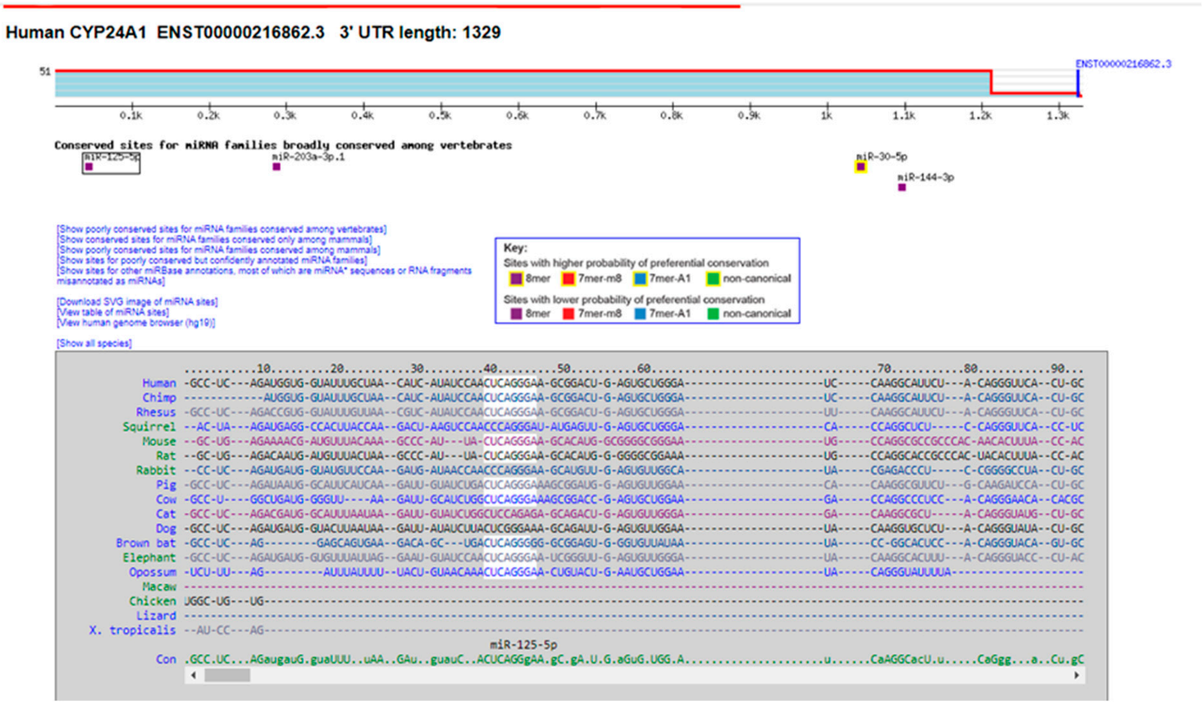

Conserved

	Predicted consequential pairing of target region (top) and miRNA (bottom)	Site type	Context++ score	Context++ score percentile	Weighted context++ score	Conserved branch length	P_{CT}
Position 40-47 of CYP24A1 3' UTR<br>hsa-miR-125a-5p	5' ...UAACAUCAUAUCCAACUCAGGGA...<br>||||||| 3' AGUGUCCAAUUUCCCAGAGUCCCU	8mer	-0.46	98	-0.46	3.280	0.68
Position 40-47 of CYP24A1 3' UTR<br>hsa-miR-4319	5' ...UAACAUCAUAUCCAACUCAGGGA...<br>||||||| 3' CACCGAAACGAGUCCCU	8mer	-0.43	98	-0.43	3.280	0.68
Position 40-47 of CYP24A1 3' UTR<br>hsa-miR-125b-5p	5' ...UAACAUCAUAUCCAACUCAGGGA...<br>||||||| 3' AGUGUUCAAUCCCAGAGUCCCU	8mer	-0.46	98	-0.46	3.280	0.68

2-CYP27B1

Human CYP27B1 ENST00000228606.4 3' UTR length: 3390

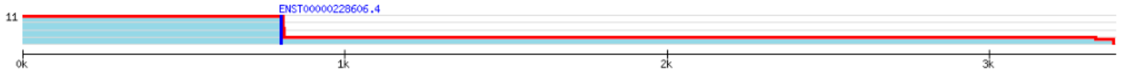

Conserved sites for miRNA families broadly conserved among vertebrates

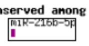

[Show poorly conserved sites for miRNA families conserved among vertebrates]
[Show conserved sites for miRNA families conserved only among mammals]
[Show poorly conserved sites for miRNA families conserved among mammals]
[Show sites for poorly conserved but confidently annotated miRNA families]
[Show sites for other miRBase annotations, most of which are miRNA* sequences or RNA fragments misannotated as miRNAs]

[Download SVG image of miRNA sites]
[View table of miRNA sites]
[View human genome browser (hg19)]

Key:
Sites with higher probability of preferential conservation
8mer 7mer-m8 7mer-A1
Sites with lower probability of preferential conservation
8mer 7mer-m8 7mer-A1

[Show all species]

```
             ......990................1000........1010......1020...........1030.....................1040..................1050................1060.
       Human --GGGAAC--------AUCCA-AGUCUAGUCUGGC--UUCCUGUAAGA--GAUU-AAGG--GGG----------UGGG---GGG---UGAAAAG----------UAAC--G--------GCAGGGGCU--AAGCU
       Chimp --GGGAAC--------AUCCA-AGUCUAGUCUGGC--UUCCUGUAAGA--GAUU-AAGG--GGG----------UGGG---GGG---UGAAAAG----------UAAC--U--------GCAGGGGCU--AAGCU
      Rhesus --GGGAAC--------GGCCA-AGUCUAGUCUGGC--UUCCUGUAAGA--GAUU-AAGG--GG-----------UGGG---GGG---UGAAAAG----------UAAC--U--------GCAGGGGCU--AAGCU
    Squirrel --GGGAAC--------AGCCA-AGUCUAGUCUUGC--UUUCAGUAAGA--GGUC-AAGG--GGG----------UGGU---AG----UGAAAAG----------UAAU--U--------GUAGGAGCU--GAGUU
       Mouse --GGGAAC--------AGCCA-AGUCUCGUCUGGC--UUUCUAGUGGA--GAUU-UAAG--GG-----------------GG----UGAACAG----------CGAG--U--------GUAGGAGCU--AAGUU
         Rat --GGGAAC--------AGCCA-AGUCCCGUCUGGC--UUCCUAGAGGG--GACU-UAAG--GG-----------------GG----CGAACUG----------UGAG--C--------GUAGGAGCU--AAGUU
      Rabbit --GGGAAG--------AGCCA-AAUUCAGGCUGGC--GUCCUGUAGGA--GAGU-AAGG--GGG----------UGGU---GC----UGAAAAG----------UGAG--C--------GCAGGGGCU--AAGCU
         Pig --GGGAAC--------AGAAA-AA-----UCCGGC--UCCCUGUAAGA--GAUU-AAGG--GGG----------UGGU---GU----UGAAAAG----------UAGC--U--------UCAACCGCU--AGGCU
         Cow ----GAAC--------AGCCA-AG-----UCUGGC--UGCCUGUAAGA--GAUU-GAGGAAGGG----------UAGU---GU----UGAAAAG----------UAGC--U--------UCAAGGGCU--AGGCU
         Cat --GGGAAU--------AGCUA-AG-----UCUGGC--UCCCUAGAAGA--GAUUUAAGG--GGG----------UGGU---GG----UGAAAAG----------UACC--U--------GCAAGGGCU--AGGCU
         Dog --GGGAAC--------AGCCAGAG-----UCUGGC--UCCCUGUAAGA--GAUU-AAGG--GGG----------UGGU---GG----UGAAAAG----------UAAC--U--------CCAAGGGCU--AGGCU
   Brown bat --GGGAAC--------AGCCA-AG-----UCUGGC--UCCCUGGAAGA--GAGU-ACGA--GGG----------UGGU---GG----UGAAAAG----------UCAC--C--------ACAAGGGCU--AG-CU
    Elephant --GAGGAC--------AGCCA-AA-----U--------------AAGA--GAUU-CAGG--GGG----------UGGU---GG----UGAAAAG----------UAAC--U--------GCAGGGGCU--GGGUU
     Opossum --GGGAGC--------AGCCA-AG-----CCUGAC--UCGCUACAAAA--AAAU-AUUA--GAG----------CAGG---AGGGCCUGAAGAAUCAGGCUU--CUAG--C--------CAAUAGGUU--AAAAA
       Macaw ------------------------------------------------------------------------------------------------------------------------------------------
     Chicken ------------------------------------------------------------------------------------------------------------------------------------------
      Lizard ------------------------------------------------------------------------------------------------------------------------------------------
X. tropicalis ------------------------------------------------------------------------------------------------------------------------------------------
                                                        miR-216b-5p
```

Conserved

	Predicted consequential pairing of target region (top) and miRNA (bottom)	Site type	Context++ score	Context++ score percentile	Weighted context++ score	Conserved branch length	P_{CT}	Predicted relative K_D
Position 1018-1025 of CYP27B1 3' UTR<br>hsa-miR-216b-5p	5' ...GUCUGGCUUCCUGUAAGAGAUUA...<br>||||||| 3' AGUGUAAACGGACGUCUCUAAA	8mer	-0.03	54	-0.01	1.955	< 0.1	-4.141

Table S2. Primers sequences for the Vitamin D metabolism genes of the related miRNAs for real-time RT-PCR analyses

Gene Name	Primer sequences 5' to 3'	
**hsa-miR-21-5p**	Left primer	GTACCACCTTGTCGGGTAGC
	Right primer	ATGTCAGACAGCCCATCGAC
**hsa-miR-216b-5p**	Left primer	GCGAAATCTCTGCAGGCAA
	Right primer	AGTGCAGGGTCCGAGGTATT
**hsa-miR-125b-5p**	Left primer	TCTGGAGTGTGACAATGGTGT
	Right primer	GCCTAGCAGTAGCTGTTTAGTGT
**hsa-miR-U6B**	Left primer	CAGCACATATACTAAAATTGGAACG
	Right primer	ACGAATTTGCGTGTCATCC
**CYP27B1**	Left primer	TACCAGAGCCTCCCGGAAC
	Right primer	AACAGCGTGGACACAAACAC
**CYP24A1**	Left primer	CCTGCTGCCAGATTCTCTGGAA
	Right primer	TTGCCATACTTCTTGTGGTACTCC
**RPLP0**	Left primer	CGACCTGGAAGTCCAACTAC
	Right primer	ATCTGCTGCATCTGCTTG
